# A Hyperspectral Survey of New York City Lighting Technology

**DOI:** 10.3390/s16122047

**Published:** 2016-12-05

**Authors:** Gregory Dobler, Masoud Ghandehari, Steven E. Koonin, Mohit S. Sharma

**Affiliations:** NYU Center for Urban Science and Progress, 1 MetroTech Center, Brooklyn, NY 11201, USA; masoud@nyu.edu (M.G.); sek9@nyu.edu (S.E.K.); mohit.sharma@nyu.edu (M.S.S.)

**Keywords:** hyperspectral, lighting technology, urban science

## Abstract

Using side-facing observations of the New York City (NYC) skyline, we identify lighting technologies via spectral signatures measured with Visible and Near Infrared (VNIR) hyperspectral imaging. The instrument is a scanning, single slit spectrograph with 872 spectral channels from 0.4–1.0 μm. With a single scan, we are able to clearly match the detected spectral signatures of 13 templates of known lighting types. However, many of the observed lighting spectra do not match those that have been measured in the laboratory. We identify unknown spectra by segmenting our observations and using Template-Activated Partition (TAP) clustering with a variety of underlying unsupervised clustering methods to generate the first empirically-determined spectral catalog of roughly 40 urban lighting types. We show that, given our vantage point, we are able to determine lighting technology use for both interior and exterior lighting. Finally, we find that the total brightness of our scene shows strong peaks at the 570 nm Na-II, 595 nm Na-II and 818 nm Na-I lines that are common in high pressure sodium lamps, which dominate our observations.

## 1. Introduction

As humanity becomes progressively more urbanized, an understanding of the urban system and the interactions between its physical components—the built infrastructures, the humans who use them and the impact of that use on the natural environment—becomes progressively more important. The human/built interface is prominent in many urban subsystems, including transportation, energy and lighting use. The latter itself has significant impact on many aspects of urban functioning: various lighting technologies have different energy consumption profiles; light pollution can have significant adverse impacts on public health, including cancer risk [1,2] and disruptions of circadian rhythms [3,4,5]; and bright city lights can disrupt avian migration [6,7].

Observations of urban lighting have been the focus of a variety of scientific inquiries with satellite observations of nighttime urban lighting in particular having a long history. One of the earliest examples of the use of satellite imaging of urban lighting at night was [8] who used imaging from LANDSAT and DMSP (the Defense Meteorological Satellite Program) to derive correlations between nighttime images and population, urban space and energy use patterns. Later, with imagery from the Operational Linescan System (OLS; a multispectral observational platform with two broad observational bands at 0.4–1.1 μm and 10.25–12.6 μm) aboard DMSP, [9] presented methods to map spatially stable visible and near-infrared emissions from cities, while [10] explored the relationship between nighttime lighting intensity and Gross Domestic Product (GDP) for over 20 countries around the world, and [11] combined DMSP-OLS imaging with detailed U.S. census data to establish a correlation with population density at more granular scales. Since those early works, there have been a plethora of studies utilizing DMSP-OLS, as well as other imaging platforms to form ever more detailed models of light-based proxies for economic activity [12,13,14,15,16,17], demographics and population estimates [18,19,20,21] and energy use [22,23,24,25].

Finally, two additional compelling use cases have emerged as the study of urban lighting via satellite imaging has evolved: lighting as a proxy for environmental impact and as a measure of urbanization dynamics. By exploiting the relationship between economic activity and carbon production, [12] utilized satellite detection of lighting to build a correlative model of carbon production based on satellite imaging, which [26] used to estimate carbon emissions at 1-km resolution from lighting data. To explore the potential for using nighttime satellite observations to study urbanization, [27,28] combined DMSP-OLS with LANDSAT Thematic Mapper imaging for the ground-truth to approximate the boundaries of diverse cities. Modern statistical techniques have been used to improve the robustness of the separation [29,30] (while updated LANDSAT data have provided enhanced ground-truth evaluation [31]), and there has been significant effort to use multi-modal observations to define urban boundaries [32,33,34]. Using long temporal baseline observations, the change in these boundaries over time has led to quantification of urban expansion in diverse areas of the globe [35,36,37,38].

While satellite observations generally give coarse views of urban lighting in both space (∼1 km for DMSP-OLS) and time, recent work has focused on remote sensing of individual sources within an urban lightscape. The work in [39] used side-facing broadband visible observations of an urban scene to identify aggregated patterns of interior lighting use in both residential and commercial buildings. With an observational cadence of 10 seconds per image, they explored the relationship between the regular macro behavior and the more random single source behavior to quantify the “pulse” of the urban system through lighting variability.

A significant advance in the study of urban lighting came from airborne observations of Las Vegas, Nevada (USA), for which [40,41] used a ProSpecTIR-VS system with a Specim AISA Eagle VNIR and Hawk SWIR. A wavelength range of 0.4–1.4 μm at 5-nm sampling was used to detect signatures of the brightest lights used in exterior illumination. These observations were correlated with the spectra of various lighting types measured in the laboratory by the National Oceanic and Atmospheric Administration (NOAA) for the NightSat mission [42], as well as satellite maps of the terrain to pinpoint specific monuments or exterior lighting sources and assign them a type. The nature of the observations did not allow for the measurements of interior lighting types, and the spatial resolution was sufficiently coarse that only the brightest lights within a given area could be identified.

In this paper, we present the first hyperspectral survey of an urban area at high spatial and spectral sampling (∼0.75 nm) utilizing a unique side-facing vantage point that allows us to quantify urban lighting at multiple wavelengths in a variety of settings. In Section 2, we present our observations and the methodology used to clean the data cubes from instrumental artifacts. In Section 4, we describe how we match the spectra of individual lighting technologies to known lighting types, as well as present a novel methodology for partitioning and mining the data to uncover unknown lighting types. We summarize our findings in Section 5.

## 2. Data Acquisition and Reduction

**Figure 1:**
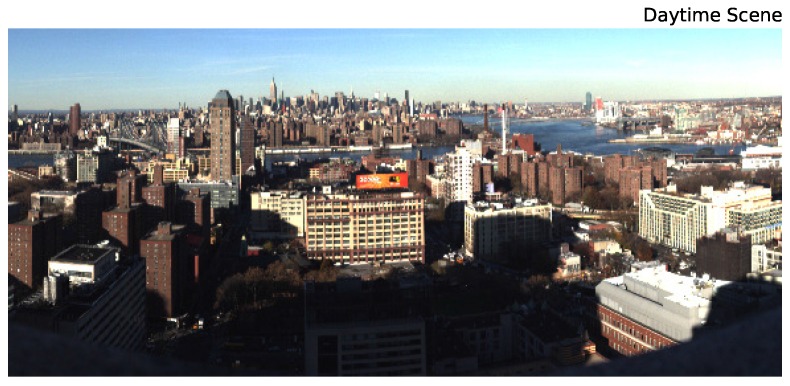
A false color image of a daytime scan of the NYC skyline with our hyperspectral instrument. For this image, RGB was mapped to (610nm,540nm,475nm). The near scene is northern Brooklyn; the mid-scene is the Manhattan Bridge and the East River; while the far scene is Midtown Manhattan.

**Figure 2:**
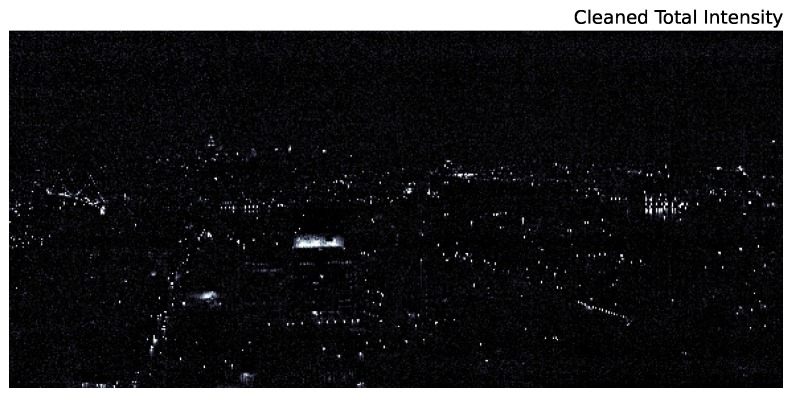
A night time version of the same scene as Figure 1 integrated from 0.4–1.0 μm.

Figure 1 shows a daytime, composite RGB image of the field of view for our hyperspectral observations. Visible in the image are Brooklyn (near-scene), the Manhattan Bridge and East River (mid-scene), as well as the Manhattan skyline (far-scene). The same scene was imaged on the night of 20 November 2013 using a Specim Ltd. ImSpector V10E Visible Near-Infrared (VNIR) hyperspectral imager provided by Middleton Spectral Vision. The nighttime scan presented in this work was a 30-s scan, and the instrument provides 872 spectral channels from 0.4–1.03 μm with a characteristic FWHM of 0.72 nanometers. The resulting field of view is roughly 75${}^{\circ }$ × 35${}^{\circ }$ with 1560 × 1600 pixels (the pixel axis ratio, determined by the scan rate, is ∼0.43). The cleaned scan is shown in Figure 2.

### 2.1. Data Reduction

**Figure 3:**
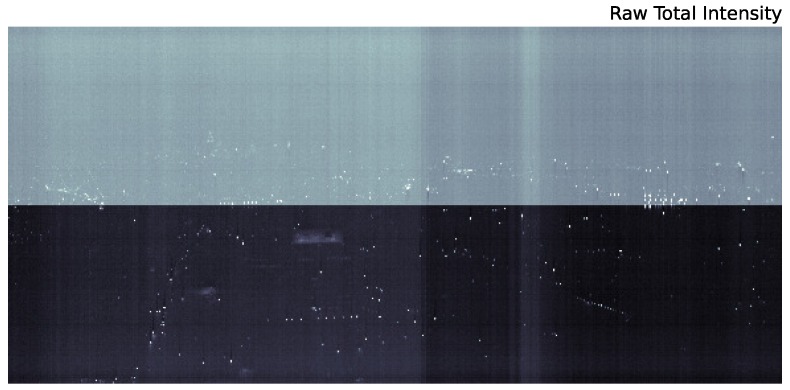
Raw data from the scan in Figure 2 integrated across the full 0.4–1.0-μm range. Artifacts due to chip offsets between the upper and lower half of the CCD, as well as gain changes during the scan (vertical stripes) are visible.

**Figure 4:**
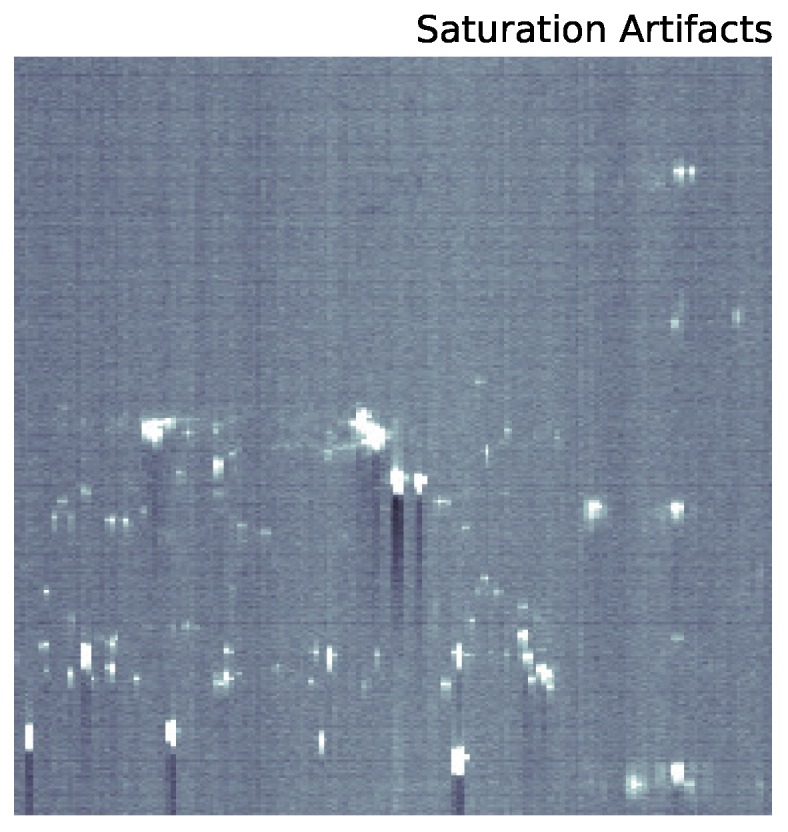
Saturation spikes as saturated sources are read off of the CCD.

Figure 2 is the result of significant processing of the raw data. These raw data, integrated across all wavelengths, are shown in Figure 3. Due to the low signal to noise of the urban scene, there are strong artifacts from several effects. First, there is an overall zero-point offset between the lower and upper half of the chip. Second, gain fluctuations as the instrument scans across the scene present as vertical features in the raw image. Finally, there are smaller scale vertical artifacts below saturated pixels (a zoom in on these features is shown in Figure 4).

**Figure 5:**
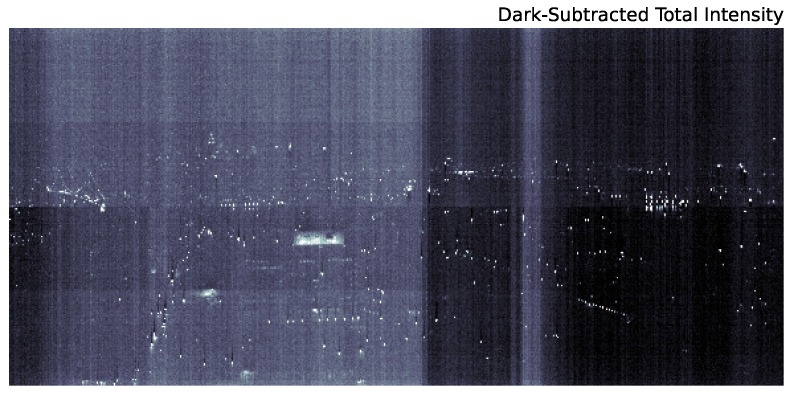
The same as Figure 3 except a dark scan has been subtracted. Although the chip offset is mostly removed, there are clearly additional chip features roughly 1/4 and 3/4 along the vertical direction of the CCD (the gain artifacts are obviously not removed by the dark scan, as well).

**Figure 6:**
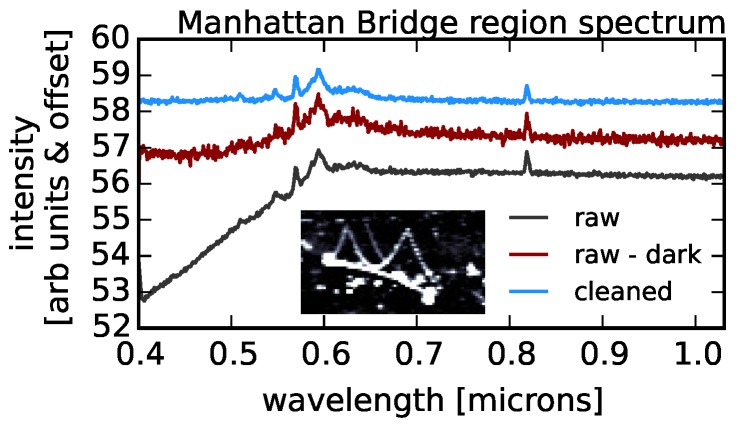
The raw, dark-subtracted (as in Figure 5) and cleaned (using the method described in Section 2) spectra of the Manhattan Bridge region shown in the inset. Large-scale, wavelength-dependent artifacts have been removed.

As a first attempt to remove these artifacts from the scan, we simply subtract a dark scan from the raw data. The result is shown in Figure 5. Although the large offset between the top and bottom of the chip is removed when subtracting the dark scan, from the figure, it is clear that there are additional chip structures roughly 1/4 and 3/4 along the vertical direction that are not captured by the dark scan. These are not gain changes along the scan since those present as vertical structures in this image. Furthermore, by subtracting an unsmoothed dark model, our cleaned image will be significantly noisier than the original. From Figure 6, it is also clear that the dark current does not adequately adjust for the decreased sensitivity at short wavelengths. Therefore, we must use an alternative method to remove image artifacts.

Our method is inspired by astronomical techniques for the removal of such features and handles both zero point offsets and gain changes along the scan direction. We perform the following steps for each wavelength. First, for each row *i*, we perform 3σ clipping by calculating the median along *i*, masking pixels, which are greater than 3σ (where *σ* is the standard deviation of pixel values along *i*), and calculating the median along *i* of the resultant values. We repeat that process 10 times and subtract the final median of *i* from each pixel in *i*. We then repeat this process across columns. The final cleaned image is shown in Figure 2. It is important to note that this method of removing artifacts relies on the image being relatively “dark”, so that a given row or column is not dominated by very bright pixels (i.e., there is a clearly-defined zero point). There is also the assumption that the background spectrum in dark regions is flat (note that ambient light pollution makes this assumption strictly incorrect; however, we find empirically that the ambient light is subdominant compared to the instrument noise).

### 2.2. Supplementary Data

**Figure 7:**
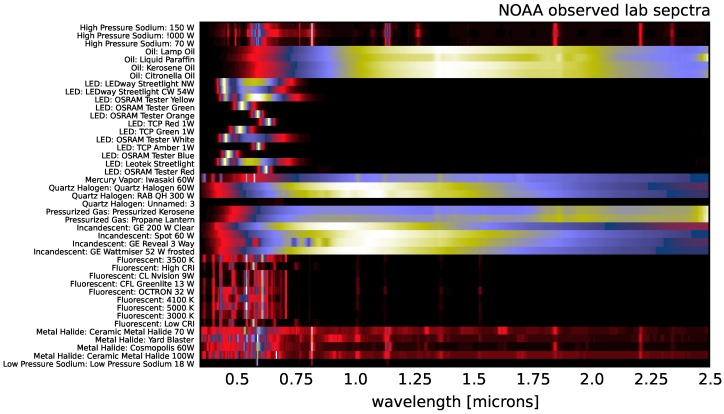
The 43 NOAA spectra measured by [43] in the lab. The wavelength range of 0.4–2.5 μm extends beyond the range of the instrument used in this work, though the resolution of one nanometer is comparable to our VNIR instrument.

In the following sections, we will also use external lighting spectral templates both for comparison of the results and direct correlation and regression. For the Nightsat Mission [42], the National Oceanic and Atmospheric Administration (NOAA) has made high resolution spectral measurements in the lab for various lighting types [43] (these templates were retrieved from [44]). These spectra span a wavelength range of 0.25–2.5 μm with a spectral sampling of one nanometer and are shown in Figure 7.

In Section 4, we will be making comparisons between our observed spectra and the NOAA template spectra. Given that our instrument and the NOAA spectra have comparable spectral resolution, we simply interpolate the NOAA templates on to our observed wavelengths as opposed to attempting to match the frequency response of the instrument used by NOAA in the lab by convolving our observations with an appropriate bandpass filter. Not only would the impact on the analysis in the coming sections be negligible, the inherent uncertainty in any convolution kernel used to match the NOAA instrument would likely not increase our accuracy.

**Figure 8:**
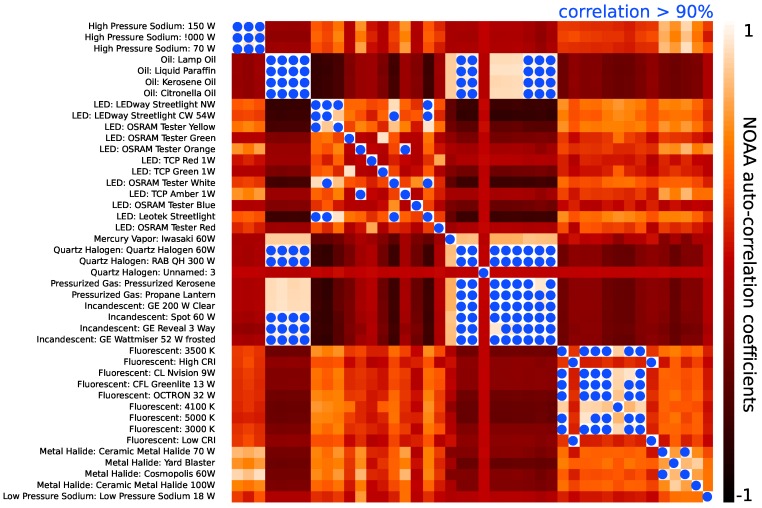
The auto-correlation matrix of the spectra shown in Figure 7. The high correlation between multiple examples of various lighting technologies (and in several cases between lighting technologies) implies that correlations with our spectra will only be able to identify type and not specific examples within a given type when correlating against the NOAA templates.

**Figure 9:**
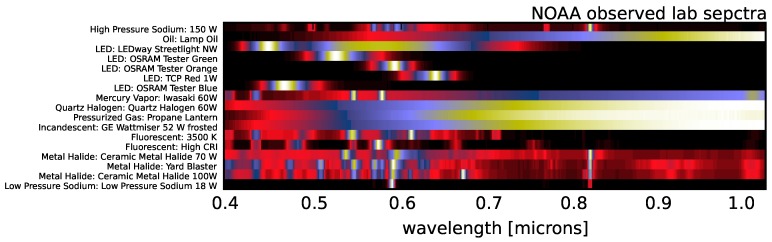
The result of pruning the spectra shown in Figure 7 to only include templates that have low co-variance. In addition, the templates have been interpolated onto the wavelengths of our VNIR instrument (see Section 2.2).

There is significant covariance between the NOAA spectra (particularly among multiple examples of a given lighting type). Figure 8 shows the auto-correlation matrix of the NOAA spectra after interpolating onto our observed wavelengths. The blue dots in the figure represent spectra that are correlated at greater than 90% and, thus, cannot be used to simultaneously tag spectra in our dataset. Some of these correlations are expected in that they are different spectra among the same lighting type; however, there are several strong correlations well off the diagonal (e.g., incandescent and quartz halogen spectra, which are strongly correlated with oil lantern spectra). We prune the templates by hand to represent the minimal set required for technology segregation. Our final list of 17 template spectra is shown in Figure 9.

## 3. Methodology

**Figure 10:**
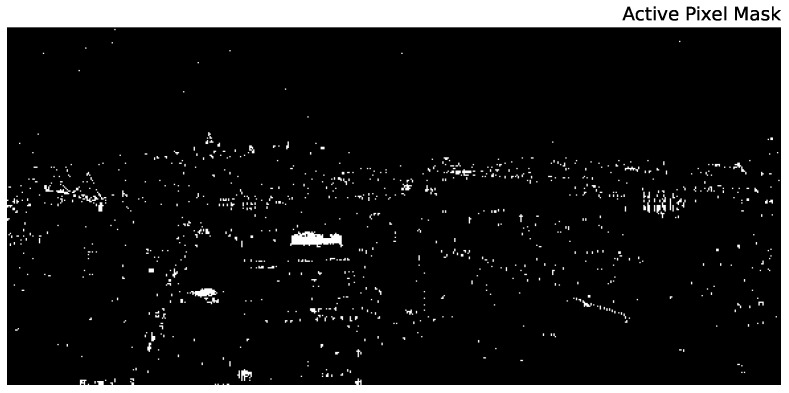
The pixels used in our analysis. This active pixel mask was created by thresholding the cleaned data and then thresholding a smoothed version of the result. The effect is to remove noise pixels from thresholding while maintaining the edges of extended structures.

For the vast majority of pixels in our data cube, the source brightness (either from direct or ambient light) is below our detection threshold. Therefore, to simplify the analysis and improve the stability of the algorithms we describe below, we create a signal mask in two steps. First, we create an initial mask of pixels for which the integrated (over wavelength) intensity exceeds an empirically-determined threshold value. Second, we then smooth that mask by a Gaussian of width 1 pixel and select only those pixels above a threshold of 0.25. This process strikes a balance between maintaining the edges of resolved sources, while removing isolated noise pixels (note that isolated signal pixels would be removed by this method, as well; however, we have manually checked that only ∼50 such pixels are removed by this process). The final mask is shown in Figure 10 and clearly includes both strong signal pixels, as well as a few pixels that are dominated by noise (e.g., visible near the top of the image). Pixels that are consistent with noise will be further pruned through various methods in our analysis below. In total, there are 41,583 “active” pixels out of 2,496,000 total pixels in the image.

We perform two distinct types of analysis: identifying spectral types that are known from laboratory measurements by NOAA and mining the observations for spectral types not in the NOAA sample. For the former, direct correlations, defined as:

(1) $$C_{i,t}=\langle s_{i}^{\prime }s_{t}^{\prime }\rangle ,$$

where:

(2) $$s_{i}^{\prime }=\frac{s_{i}-\langle s_{i}\rangle }{\sigma_{i}},$$

$s_{i}$ is the intensity as a function of wavelength for pixel *i*, $s_{t}$ is the intensity as a function of wavelength for NOAA spectral template *t*, $\sigma_{i}$ is the standard deviation of pixel *i* and the averages are over the wavelength, with the NOAA spectral templates suffices to identify pixels with high correspondence with known templates. For the latter, we use a variety of unsupervised machine learning and data mining techniques. Unsupervised learning has a long history of application in remote sensing in a variety of contexts (e.g., see [45,46,47]); however, this is the first time these methods have been applied systematically to lighting technology identification.

### 3.1. k-Means Clustering

Our first method for separating lighting types is to cluster signal pixels according to their spectral characteristics using *k*-means clustering [48,49,50]. The procedure first chooses *k* random cluster centers (i.e., random spectra) and then, for each pixel, associates that pixel with the nearest cluster center. Here “nearest” is defined as the smallest sum of squared differences,

(3) $$d_{i,j}={\left(\sum_{m}{\left(s_{i}\left(\lambda_{m}\right)-c_{j}\left(\lambda_{m}\right)\right)}^{2}\right)}^{1/2},$$

where $s_{i}\left(\lambda \right)$ and $c_{j}\left(\lambda \right)$ are the *i*-th pixel spectrum and the *j*-th cluster center (respectively) at wavelength *λ*. Each cluster center is then updated to be the mean of all $s_{i}\left(\lambda \right)$ associated with that cluster. The procedure is repeated until the cluster centers converge. The number of cluster centers must be set by hand prior to initialization.

### 3.2. Template-Activated Partition Clustering

Given the large sample size (∼42,000 spectra), it is unlikely that *k*-means will yield all observed lighting types for *k* on the order of 10. We could increase *k* significantly (with corresponding increases in computation time), but this would likely still miss small clusters (i.e., lighting types with only a few examples in the observations), and it is not clear that the distance-based *k*-means algorithm is optimal for returning “all” clusters in the data.

In fact, we note that this is an extremely difficult scenario for clustering in general for several reasons. First, the dimensionality is very high ($N_{\dim}=872$, the number of spectral channels). Second, the number of clusters is unknown. Third, the clusters themselves are not of uniform size in that different lighting types may have different spectral variability among instances. Fourth, the clusters are not uniformly sampled as there is no reason to expect that each lighting type has the same number of instances in our data. Finally, the last two imply that the cluster density is non-uniform.

Different clustering methods are useful in various scenarios (e.g., DBSCAN [51] excels at finding uniform density clusters in data), and so, we now use a combined approach of multiple clustering schemes in parallel. In particular, we use *k*-means, DBSCAN and hierarchical clustering [52] in tandem and filter the results to produce a reasonably orthogonal set of observed lighting types.

**Figure 11:**
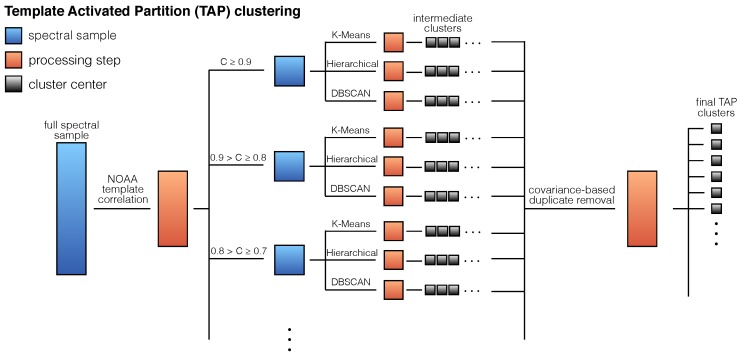
An illustration of the Template-Activated Partition (TAP) clustering approach. From left to right, the full spectral sample is partitioned into subsets according to the correlation with NOAA templates, each subset is independently clustered by three unsupervised clustering methods, and the results are pooled with duplicates removed to form the final TAP cluster centers.

The analysis proceeds in three steps and is illustrated in Figure 11. First we partition the data using correlations with the NOAA templates. That is, we break the parent sample of 41,583 pixels into 10 subsamples, one that contains all spectra with $\max_{t}\left(C_{i,t}\right)\in \left(0.9,1.0\right]$, one that contains all spectra with $\max_{t}\left(C_{i,t}\right)\in \left(0.8,0.9\right]$, ..., and one that contains all spectra with $\max_{t}\left(C_{i,t}\right)<0.1$. This Template-Activated Partition (TAP) is useful since we know that some of the NOAA templates are well represented and form significant clusters, but there are also minor variations (e.g., line ratios), which may signal different lighting types; so, those may be easiest to extract if clustered independently. In the second step, we perform the three clustering methods described above in parallel (the three clustering methods are carried out using the Scikit-learn package [53]), generating a set of cluster centers for each of the partitions and for each of the clustering methods. Finally, we filter out duplicates among all of those cluster centers by thresholding the covariance among them.

## 4. Results

### 4.1. Correlation with Known Templates

**Figure 12:**
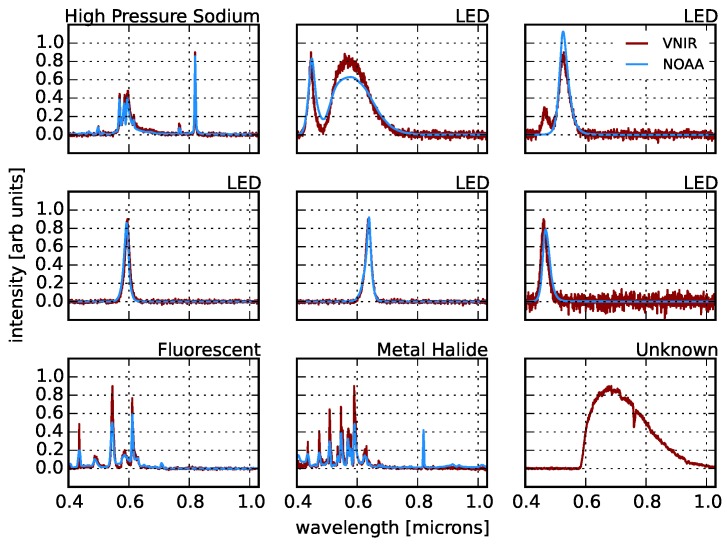
Several examples of high S/N spectra in our sample that are correlated with the NOAA templates shown in Figure 9. The bottom right spectrum is an example of a lighting technology that is not represented in the lab templates.

**Figure 13:**
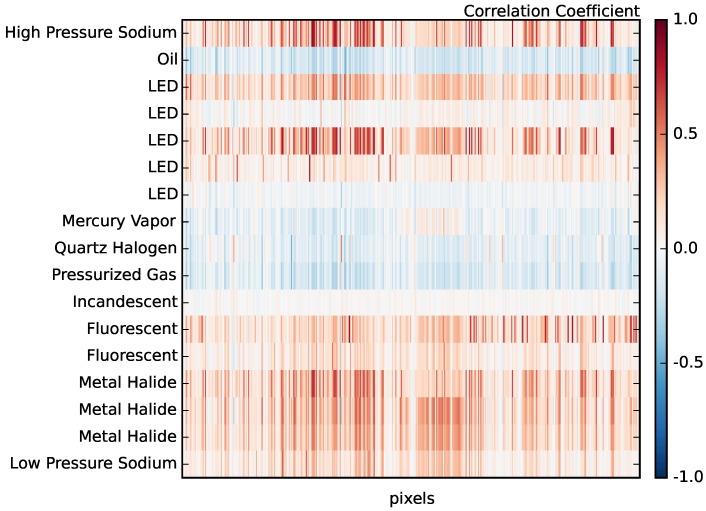
The correlation coefficients for all 41,583 spectra in our dataset with each of the NOAA templates in Figure 9. We clearly detect examples of 13 of the templates with high correlation coefficients. Interestingly, we do not find examples of incandescent light bulbs (see Section 4.5).

In Figure 12, we show example pixel spectra from our scan, both for spectra that clearly correspond to the spectra measured by NOAA in the lab and also one example of a spectrum that is not in the NOAA catalog. In particular, we find high correlations (>85%) with High Pressure Sodium (HPS) lamps, various LEDs, fluorescents and metal halide lights. Figure 13 shows the correlations coefficients for all active pixels and for all NOAA templates. In our sample, we find that HPS, LED, fluorescent, metal halide and Low Pressure Sodium (LPS) lights make up the dominant lighting in our scene, while oil lamps, mercury vapor, pressurized gas, incandescent and quartz halogen lamps are largely undetected.

### 4.2. Unsupervised Learning

As we have seen from Figure 12 and Figure 13, while several of the NOAA lighting types are detected in our scene with high confidence, many of the pixels do not correspond directly to one of those spectral templates. Nevertheless, it is reasonable to assume (and we shall see that it is indeed the case) that not all pixel spectra that are uncorrelated with the NOAA templates ($C_{i,t}<85\%$ for all *t*) are unique. Rather, there are likely lighting types that are present in our sample, but not in the NOAA templates. However, there are two important unknowns, the overall number of lighting types and the number of instances of each type. We will first estimate the total number of types using unsupervised clustering techniques and then classify our pixel spectra among those.

#### 4.2.1. *k*-Means Clustering

**Figure 14:**
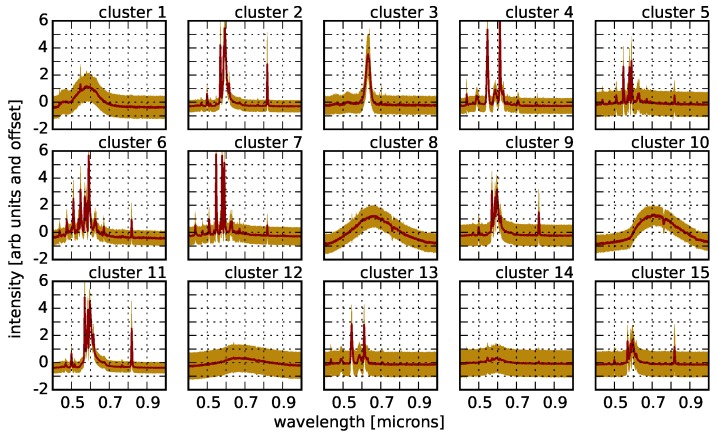
The result of clustering our ∼42,000 spectra into 15 clusters using *k*-means clustering. The red lines represent the cluster centers, while the yellow bands represent the dispersion of the cluster members for each cluster. It is clear that *k*-means is recovering dominant spectra for High Pressure Sodium (HPS) lamps, as well as fluorescent and metal halide lamps (see Figure 15). Nevertheless, several of the cluster centers do not correspond to any NOAA templates, but are largely consistent with LED-type lights.

**Figure 15:**
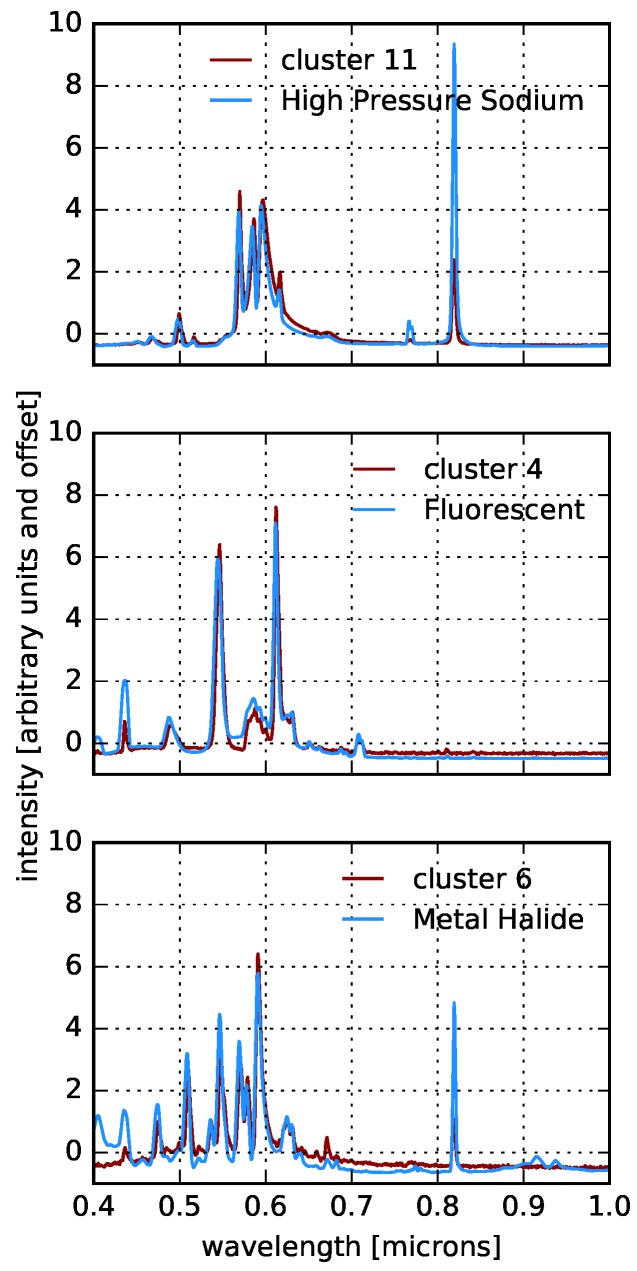
A comparison of several of the *k*-means cluster centers from Figure 14 with NOAA templates.

The results of applying *k*-means clustering to our active pixels using k=15 are shown in Figure 14. Varying the number of *k* to be fewer or more than 15 does not result in qualitatively different cluster centers. This first pass at clustering is clearly identifying distinct lighting types that are associated with previously known spectra (see Figure 15), as well as types that are not among the NOAA templates. There are also “duplicates” among the cluster centers that represent the same type, but with minor variations in line ratios or continuum shape. We note that several of the new lighting types identified in Figure 14 are qualitatively similar to NOAA LEDs, and so, we tentatively identify their type as such, but caution that without the ground-truth, we cannot be certain.

**Figure 16:**
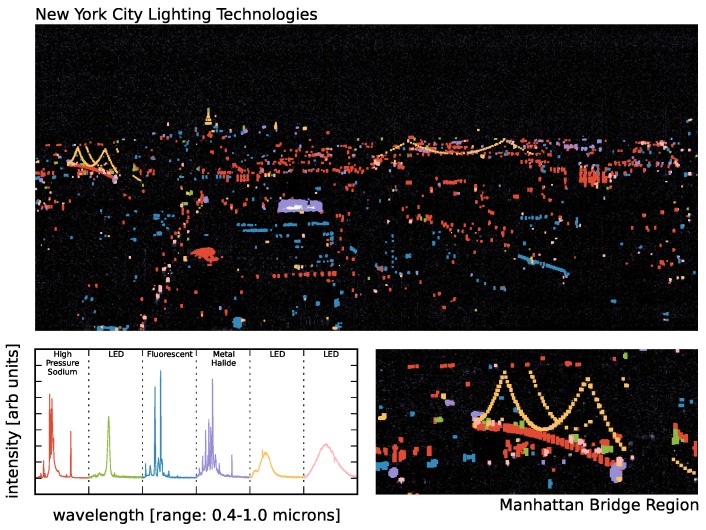
A projection of the cluster tags for several cluster centers on the integrated image of Figure 2 (**top**). The associated spectra (with corresponding colors) are shown in the **lower left**. A zoom-in of the Manhattan Bridge region is shown in the **lower right** demonstrating that detailed lighting technology use can be identified with our methodology.

For lighting technologies, the ground-truth we need can come in the form of publicly available records’ data. We show an example in Figure 16. The associated active pixels for six lighting types are shown in a zoom-in of the Manhattan Bridge. The HPS, fluorescent and metal halide types clearly correspond to the NOAA templates, as does one of the LED types. The remaining two LED types are assumed to be LEDs based on their similarity to the LEDs in the top row of Figure 12. The image shows that the necklace lights of the Manhattan Bridge all correspond to the same cluster, which is presumed to be an LED. We confirm that this is indeed the case as the necklace lights of the Manhattan Bridge underwent a retrofit to LEDs shortly before our VNIR scan was taken [54]. We also point out that the lights along the bridge are HPS lamps and have been labeled by our clustering as such, while the head and tail lights of traffic along the bridge are labeled as LEDs. The figure also shows the full tagged image including the LED necklace lights on the Williamsburg bridge that were retrofitted at the same time as the Manhattan Bridge. Additional important features are the metal halide spotlights lighting the billboard near the center-left of the image, the different color LEDs in the roof versus the spire of the Empire State Building, the Chrysler building LEDs and the fluorescent interior lights in the office building directly under the billboard.

#### 4.2.2. Template-Activated Partition Clustering

**Figure 17:**
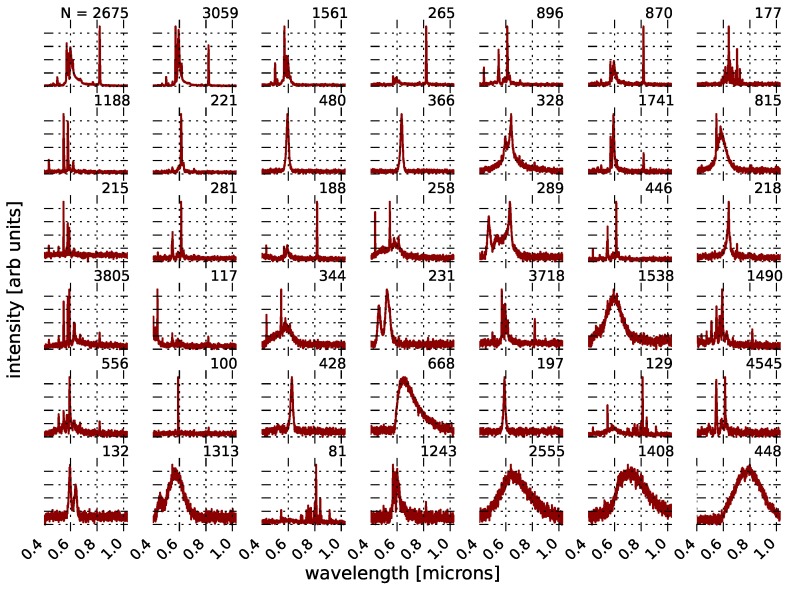
The results of performing template-activated partition clustering (see Figure 11 and Section 4.2.2) on our dataset to identify all statistically-robust observed lighting types. The methods of clustering used were *k*-means, DBSCAN and hierarchical.

While performing *k*-means on the full sample of ∼42,000 spectra has successfully identified characteristic lighting types that are both known (from NOAA templates) and new, as noted in Section 3.2, clustering on samples of this size is not likely to generate the full set of observed lighting types with k=15. Applying TAP clustering to this sample produced 42 independent clusters in the data, which are shown in Figure 17. While some of these are obviously those lighting types that are represented by the NOAA templates, there are many that are not. For example, the fourth row, second column is a cluster of 117 lights that are very bright in the far blue, but otherwise dark, while the bottom row has examples of lights with both broad and narrow line features that are beyond the human vision limit of 700 nanometers and are strongly peaked in the NIR.

These 42 different lighting types represent the first catalog of empirically-measured urban lighting technology. While incomplete, these lighting types are potentially useful in a number of urban studies, and so, we make the cluster centers, as well as the cleaned, observed spectra publicly available [55].

### 4.3. Aggregate Spectrum

**Figure 18:**
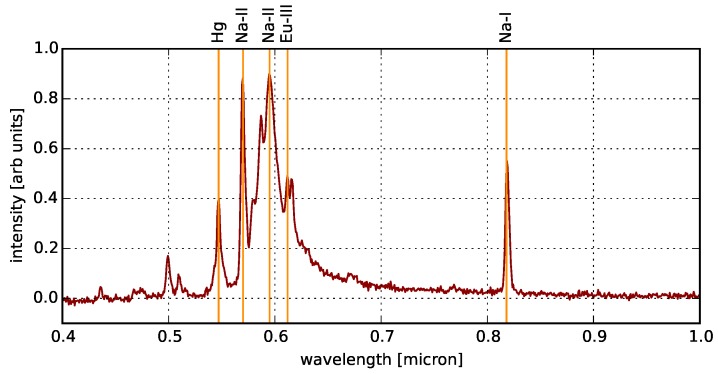
The aggregate spectrum of *all* pixels in our scene. The integrated spectrum of the NYC skyline is dominated by HPS-type lighting with strong peaks in the Na-I and Na-II bands, though we also identify Hgand Eu-III lines that are common in fluorescent lighting.

The aggregate spectrum for all pixels in our scene is shown in Figure 18. By comparing with the template spectra in Figure 12, we find that the scene is dominated by HPS lamps; and indeed, Figure 17 shows that HPS-type spectra are the most common in our dataset. In fact, the brightest lines in the aggregate spectrum are clearly Na-I and Na-II, though we also see the Hg and Eu-III lines common in fluorescent lighting.

### 4.4. Applications

The sensitivity and spatial and spectral resolution of the instrument, coupled with our ability to distinguish both interior and exterior lighting technologies suggest several potential applications of our methodology. First, with persistent scanning, we can not only measure the time-dependent brightness of the city at high spectral resolution (cf. [56] for a low spectral resolution example) for correlation with data from migratory bird strikes and human circadian rhythm disruptions (e.g., [39]), but we can assess changes in the lighting technology use over time. For example, NYC Local Law 84 requires that all large tenant spaces convert to energy-efficient lighting by 2025. In addition, given the synoptic nature of our side-facing vantage point, this technology can identify buildings for energy-efficient lighting upgrades (as in [57], but for many buildings simultaneously). Finally, since our observations of the skyline also include coverage of the sky above the city, we can assess the detailed wavelength-dependent reflectivity and amplification of artificial light by cloud cover [58,59].

### 4.5. Discussion

Despite the success of our methodology in differentiating various lighting technologies, there are several factors to consider before interpreting the resultant spectra. First, we note that one of the most important aspects of this work is that we have demonstrated the ability to identify lighting type for both exterior and interior lighting. For example, the rows of fluorescent lights just left of center in Figure 16 are interior lights from an office building on New York University’s Tandon School of Engineering campus. While there is tremendous potential for these observational techniques as a tool to quantify technology penetration in cities, it also implies that curtains, interior reflections and mixed lighting type use (all of which are wavelength dependent) can complicate the interpretation. For the first two, sharp features in the spectra mitigate this effect to a certain extent since curtains and reflections likely have broadband impact, while for the latter, in future work, we will use component separation methods to model each lighting pixel as being mixed use.

The second complicating factor is that, although we have sufficient signal to noise in this scan to identify exterior lighting type as distant as 4–5 miles for bright building lights, for interior lights, the signal amplitude is low enough that only interior lights within about one mile are visible. Within that distance, there are no detectable incandescent light bulbs, for example. To achieve sufficient signal to noise to identify interior lighting technologies at greater distances, future work will use stacks of multiple scans.

Finally, we point out that the instrument itself does not completely cover the horizontal area across which it is scanning. As the instrument pans, it exposes the CCD at a specified frame rate, and so, horizontal areas that are scanned between exposures are not captured, potentially leading to completely missed lights. In future work, we will mitigate this effect by reducing the scan rate and exposure time and integrating pixels along the scan direction.

## 5. Conclusions

We have presented the first ground-based hyperspectral observations of a city at night for the purpose of empirically determining exterior and interior lighting technology usage. Our observations were carried out with a Visible Near-Infrared (VNIR) 0.4–1.0-μm single slit scanning spectrograph with 872 spectral channels. We have shown that this spectral resolution can easily distinguish lighting of various types when comparing against spectral templates generated with laboratory measurements by the National Oceanic and Atmospheric Administration (NOAA). Furthermore, the resolution and sensitivity of the instrument allows for the determination of unknown lighting types using unsupervised machine learning and data mining techniques.

Our observations are brightness limited, and by correlating pixels with external NOAA templates, we find that the dominant lighting source visible within our field of view of the New York City skyline is HPS lamps. Simple *k*-means clustering in $N_{\dim}=872$ dimensions of the full dataset yields cluster centers (i.e., characteristic spectra) that, in some cases, correspond to the previously known spectral templates, while in other cases representing the identification of unknown lighting types. We have used observations of the Manhattan Bridge to ground-truth and validate our unsupervised classification by noting that our algorithm correctly identifies the bridge necklace lights as LEDs and the road lighting as HPS lamps.

We have attempted to extract as many lighting types as possible using TAP clustering. By partitioning the data into subsets based on the amplitudes of correlation with known templates and then independently clustering those subsets with a variety of unsupervised learning methods, we have identified approximately 40 different characteristic spectra (lighting types) in our scene. The clustering methods we used for the subsets (*k*-means, DBSCAN and hierarchical) were performed in parallel and then combined to yield the final catalog. By using TAP clustering to segment the data and then multiple clustering techniques that incorporate different metrics to determine cluster centers, we address the fact that clusters of lighting technologies in this space are of unknown number, of variable size, non-uniformly sampled and of variable density. Furthermore, the partitioning ensures that lighting types that have spectra with minor deviations (e.g., line ratios, continuum variations, etc.) are captured by the clustering.

One of the great promises of this methodology is to use correlative records’ data to measure the relationship between urban populations and technology by empirically measuring interior lighting usage in commercial and residential scenes and correlating those with factors such as socio-economic status, demographics, business type and building age. With the present scan, we are able to detect interior fluorescent lighting for nearby (Downtown Brooklyn) office buildings, but do not have a sufficient signal to noise to detect interior lighting in the distant scene (Manhattan). However, in future work, we will stack multiple scans of the same scene to boost the signal to noise for accurate determination of interior lighting types, as well as the spectral content of diffuse light pollution in New York City.
